# Characteristic and Mechanism of Drug-Herb Interaction Between Acetylsalicylic Acid and Danhong Injection Mediated by Organic Anion Transporters

**DOI:** 10.3389/fphar.2020.577012

**Published:** 2020-10-02

**Authors:** Jianping Li, Jingbo Lu, Yin Peng, Xuejun Xu, Chenkai Chen, Ming Gao, Ling Lin, Jianming Guo, Jinao Duan

**Affiliations:** ^1^Jiangsu Collaborative Innovation Center of Chinese Medicinal Resources Industrialization, Nanjing University of Chinese Medicine, Nanjing, China; ^2^Jiangsu Key Laboratory for High Technology Research of TCM Formulae, Nanjing University of Chinese Medicine, Nanjing, China; ^3^Pharmaceutical Department, East Region Military Command General Hospital, Nanjing, China

**Keywords:** organic anion transporter, drug interaction, acetylsalicylic acid (aspirin), Danhong injection, salicylic acid, aristolochic acid

## Abstract

The mixture of *Salvia miltiorrhiza* and *Carthamus tinctorius* (Danhong injection, DHI) is widely prescribed in China for the treatment of cardiovascular and cerebrovascular diseases. In most cases, DHI is used in combination with acetylsalicylic acid (aspirin, ASA). However, the interaction between DHI and ASA remains largely undefined. The purpose of this study is to explore the interaction profile and mechanism between DHI and ASA. The frequency of drug combination of DHI and ASA was analyzed based on 5,183 clinical cases. The interaction characteristics were evaluated by analyzing the pharmacokinetics and disposition profile of salicylic acid (SA, the primary metabolite of ASA) in rats. The interaction mechanisms were explored through evaluating the hydrolysis of ASA regulated by ASA esterase, the tubular secretion of SA mediated by influx and efflux transporters, and the tubular reabsorption of SA regulated by urinary acidity-alkalinity. The inhibitory potential of DHI on organic anion transporters (OATs) was further verified in aristolochic acid I (AAI) induced nephropathy. Clinical cases analysis showed that DHI and ASA were used in combination with high frequency of 70.73%. In drug combination of DHI and ASA, the maximum plasma concentration of SA was significantly increased by 1.37 times, while the renal excretion of SA was significantly decreased by 32.54%. The mechanism study showed that DHI significantly inhibited the transport function, gene transcription and protein expression of OATs. In OATs mediated AAI nephropathy, DHI significantly reduced the renal accumulation of AAI by 55.27%, and alleviated renal damage such as glomerulus swelling, tubular blockage and lymphocyte filtration. In drug combination of DHI and ASA, DHI increased the plasma concentration of SA not through enhancing the hydrolysis of ASA, and the tubular reabsorption of SA was not significantly affected. Inhibition of tubular secretion of SA mediated by OATs might be the reason that contributes to the decrease of SA renal excretion.

## Introduction

Drug-drug interaction (DDI) refers to the pharmacokinetics and pharmacodynamics characteristics alteration of one drug induced by the presence of other drugs. Unanticipated DDIs are suggested to be the major causes of uncontrollable efficacy and safety issues associated with prescription drugs. For example, probenecid, one of the mostly recognized inhibitors of organic anion transporters (OATs), would delay the OATs mediated tubular secretion of penicillin and thus enhance the antibacterial effect of penicillin (Robbins et al., 2012). Conversely, mibefradil, a calcium channel blocker, has been withdrawn from the market because of its potent inhibitory effect on cytochrome P450 that leads to severe clinical risks of rhabdomyolysis during its concurrent treatment with simvastatin (Schmassmann-Suhijar et al., 1998). Therefore, it is important to understand the profile and mechanism of DDIs in order to ensure the drug safety and efficacy.

Acetylsalicylic acid (aspirin, ASA) is a representative antiplatelet drug and is widely prescribed to prevent first-time cardiovascular disease in high-risk patients at low daily doses. ASA could also be used as a classic analgesic and anti-inflammatory agent at higher daily doses. Danhong injection (DHI) is the aqueous extract of two Chinese medicines, the radix and rhizome of *Salvia miltiorrhiza Bunge* (Labiatae) and the dry flower of *Carthamus tinctorius L*. (Asteraceae). DHI is widely prescribed in China for the treatments of cardiovascular and cerebrovascular disease such as myocardial infarction and cerebral thrombosis. Salvianolic acids such as lithospermic acid (LA), protocatechuic aldehyde (PA), salvianolic acid A (SaA), salvianolic acid B (SaB), rosmarinic acid (RA), tanshinol (DSS), and caffeic acid (CA) are primary components of DHI (Liu et al., 2013; Li et al., 2016a).

DHI and ASA were widely used in combination in clinic for the treatment of cerebral infarction and coronary heart diseases (Du et al., 2011), it is important to determine whether there is an herb-drug interaction between DHI and ASA, and whether this interaction potential would influence the drug efficacy and safety. Our previous studies have revealed the herb-drug interaction between DHI and ASA by metabolomics strategy and multivariate statistical analysis (Li et al., 2016b), and have suggested that DHI could alleviate ASA-induced gastric mucosal damage during drug combination of DHI and ASA (Li et al., 2016a). The present study aimed to evaluate the herb-drug interaction profile between DHI and ASA by pharmacokinetics analysis, and to explore the mechanisms of potential interactions.

## Materials and Methods

### Materials and Animals

ASA (purity ≥ 99%) was purchased from Aladdin Chemistry Co., Ltd. (Shanghai, China). Standards of salicylic acid (SA), SaA, SaB, RA, DSS, PA, CA, and LA (HPLC ≥ 98%) were purchased from Chinese Materials Research Center (Nanjing, China). Aristolochic acid (AAI), estrone sulfate (ES), para-aminohippurate (PAH), and probenecid (purity ≥ 99%) were purchased from Sigma-Aldrich Co. LLC. (St. Louis, USA). Liver microsome of male Sprague-Dawley (SD) rat was purchased from Research Institute for Liver Disease Co., Ltd. (Shanghai, China). Transgenic HEK293 cell lines stably overexpressing human *OAT1* (Cat. No. GM1003) and *OAT3* (Cat. No. GM1004) were supplied by GenoMembrane Lo. Ltd. (Yokahama, Janpan).

DHI was provided by Buchang Pharma Co., Ltd., China (Shandong, China; Lot Number: 15081038). Chromatographic fingerprint of DHI was established by UHPLC-PDA-QTOF/MS analysis (**Figure S1**). Seven peaks were identified as SaA, SaB, RA, DSS, PA, CA, and LA by comparing retention time, UV spectrum and MS fragment with corresponding standards (**Figure S2**). SaA, SaB, RA, DSS, PA, CA, and LA are primary components of DHI, and the concentrations of SaA, SaB, RA, DSS, PA, CA, and LA in DHI were 41.73 ± 1.83, 410.42 ± 4.91, 21.40 ± 0.32, 173.98 ± 5.34, 24.37 ± 0.41, 1.03 ± 0.01, and 1.17 ± 0.02 μg/ml, respectively.

Specific Pathogen Free (SPF) male SD rats weighting 350–400 g and male C57BL/6 mice weighting 25–30 g were purchased from Vital River Laboratory Animal Technology Co. Ltd. (Beijing, China; License Number: SCXK (Jing) 2012-0001). All animals were kept in the Drug Safety Evaluation Center of Nanjing University of Chinese Medicine (Nanjing, China). Experiments conducted on animals were approved by the Animal Ethics Committee of Nanjing University of Chinese Medicine, and performed in compliance with the Guide for the Care and Use of Laboratory Animals.

### Analysis of Clinical Cases

Clinical cases of DHI in Nanjing General Hospital of People’s Liberation Army from July 2012 to July 2017 were collected, and the cases with missing information such as medication time, administration dosage and combination drugs were excluded. The drugs that used in combination with DHI were placed in order according to their number of cases. The time interval between DHI and the drug that most commonly used in combination with DHI (i.e. ASA) was further analyzed. The results obtained from clinical case analysis were prepared for the further experiment design of herb-drug interaction between DHI and ASA that conducted on rats.

### Experimental Design of Drug-Herb Interaction Between DHI and ASA

**Figure 1:**
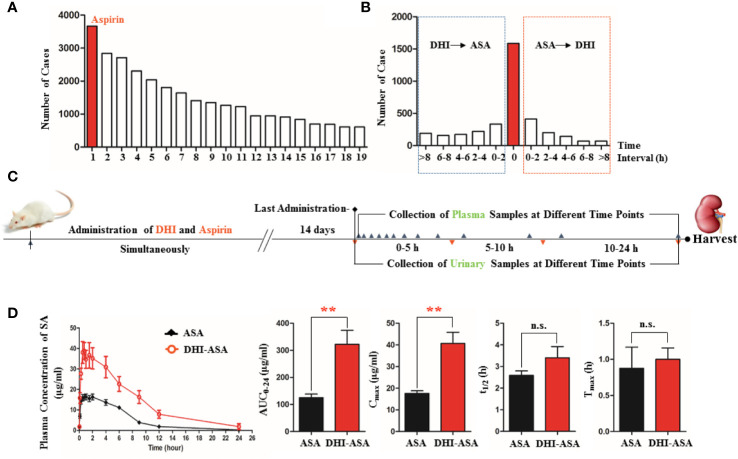
Altered pharmacokinetics profile of SA during drug combination of DHI and ASA. **(A)** Drugs that commonly used in combination with DHI in clinic (1. ASA, 2. Clopidogrel, 3. Oxiracetam injection, 4. Atorvastatin calcium, 5. Mecobalamin injection and tablet, 6. Rosuvastatin calcium, 7. Heparin sodium injection, 8. Lidocaine injection, 9. Fasudil injection, 10. Phenobarbital injection, 11. Amlodipine, 12. Troxerutin injection, 13. Vitamin B1, 14. Edaravone injection, 15. Alprostadil injection, 16. Pantoprazole injection, 17. Potassium chloride sustained-release tablet, 18. Butylphthalide soft capsule, 19. Vitamin B6); **(B)** Time interval of drug combination of DHI and ASA in clinic; **(C)** Experimental design of herb-drug interaction between DHI and ASA conducted on rats; **(D)** Pharmacokinetic profile and parameters of SA. **P < 0.01.

Twelve male SD rats weighting 350–400 g were randomly divided into two groups, rats in Group ASA were administrated with ASA solution (10.41 mg/kg) intragastrically; rats in Group DHI-ASA were injected with DHI (4.16 ml/kg) through caudal vein immediately after the administration of ASA solution (10.41 mg/kg). All rats were administrated daily at scheduled time (9:00–10:00 a.m.) for 14 consecutive days (**Figure 1C**). In this study, a total of two cohorts of animal experiment were performed, one cohort was for the evaluation of pharmacokinetic profile of SA, and the other was for the assays of renal excretion of SA, pH value of urine, gene transcription, and protein expression of OATs.

Blood was sampled from retrobulbar venous plexus of each rat at different time points (1, 5, 15, 30, 45 min, 1, 1.5, 2, 4, 6, 9, 12, and 24 h after the last administration). The blood volume of each collection was 0.3 ml, and the blood was placed in a PE tube containing 33 μl of sodium citrate solution (3.8%, w/v). During the process of blood sampling, physiological saline was injected intraperitoneally every two hours to maintain the circulating blood volume of rats (Diehl et al., 2001). The blood samples were centrifuged at 13,000 rpm/min for 10 min to collect plasma. An aliquot of 100 μl plasma was pipetted into a new PE tube, and mixed with 100 μl of internal standard solution (diphenhydramine, 0.71 μg/ml) and 200 μl of methanol thoroughly. The mixture was centrifuged (13,000 rpm/min, 10 min) to obtain the supernatant for determination of SA concentration by UHPLC-MS/MS analysis.

### Determination of Inhibition Potential of DHI on the Activity of ASA Esterase

To determine the effect of DHI on the activity of plasma ASA esterase *in vivo*, twelve male SD rats weighting 350–400 g were randomly divided into two groups, rats in Group CTL were supplied with food and water regularly; rats in Group DHI were injected with DHI (4.16 ml/kg) through caudal vein daily for 14 consecutive days. On the 15^th^ day, blood was sampled from the retrobulbar venous plexus of each rat, and mixed with sodium citrate solution (3.8%, w/v) to obtain plasma. An aliquot of 100 μl plasma were pipetted into a new PE tube, and mixed with 10 μl of ASA hydrochloride solution (100 mM) and 890 μl of Tris buffer (pH 7.4) thoroughly. The mixture was incubated at 37°C in a water bath for 20 min. Then an aliquot of 100 μl reaction solution was mixed with an equal volume of internal standard solution (diphenhydramine, 0.71 μg/ml) thoroughly to stop the reaction and to precipitate the protein. The mixture was centrifuged at 13,000 rpm/min for 10 min to obtain the supernatant for determination of SA concentration by UHPLC-MS/MS. One unit of ASA esterase activity (U) was defined as the production of 1 μmol of SA in a reaction system with the substrate concentration of 1 mM, pH of 7.4 and temperature of 37°C per minute and per milliliter of plasma. Furthermore, to clarify the relationship between the plasma concentration of DHI and the inhibition effect on ASA esterase, rats in Group DHI were injected with DHI (4.16 ml/kg) through caudal vein after the first blood collection, and plasma was sampled at different time points (1, 15, and 60 min after the single injection of DHI) for determination of the activity of ASA esterase.

To determine the effect of DHI on the activity of plasma ASA esterase *in vitro*, a total of 1.8 ml fresh blank plasma of male SD rat was divided into 18 aliquots. The aliquots were randomly divided into three groups as follows (n = 6): Group CTL, no DHI was added to the reaction system; Group DHI-1, 10 μl of DHI was added to the system and the final concentration of DHI was 100-fold dilution of the original DHI; Group DHI-2, 100 μl of DHI was added to the incubation system and the final concentration of DHI was 10-fold dilution of the original DHI. Furthermore, to determine the effect of DHI on the activity of ASA esterase in liver microsome, a total of 450 μl of blank liver microsome of male SD rat was divided into three group as that in the assay of plasma ASA esterase (five aliquots of 30 μl in each group, n = 5). The activity of ASA esterase in plasma and liver microsomes was determined according to the method detailed above *in vivo* study.

### Determination of SA Renal Excretion and Urinary pH Value

Rats were housed in metabolism cages immediately after the last administration, and urine samples of each rat were collected on ice at different time periods (0–5, 5–10, and 10–24 h). The urine samples were centrifuged at 3,000 rpm/min for 10 min to precipitate impurities, and the urinary pH value was detected by pH meter. Meanwhile, an aliquot of 400 μl supernatant was mixed with 100 μl of methanol thoroughly to precipitate some protein, and the final supernatant was obtained for the determination of SA concentration by UHPLC-MS/MS analysis.

### Determination of the Effect of DHI on Gene Transcription and Protein Expression of OATs

Rats were sacrificed after the last administration, and kidneys of each rat were sampled for qPCR and Western blot analysis to evaluate the gene expression and protein levels of OAT1, OAT2, and OAT3, respectively. The primer sequences of *rOAT1*, *rOAT2*, and *rOAT3* were detailed in **Table 1**.

**Table 1:** Primer sequences of rat OATs (*rOAT1*, *rOAT2*, and *rOAT3*) and human OATs (*hOAT1*, *hOAT2*, and *hOAT3*).

Primer	Sequence (5’-3’)
***rOAT1***	**(S)** TCAACTGCATGACACTAAATGTG; **(AS)** AGCCTTCCTGAGGAGGAGTA
***rOAT2***	**(S)** ATCATTGTGCTCCCACTGGAG; **(AS)** CTCCACACGACCCTGGGTTA
***rOAT3***	**(S)** CAGCATCTCGGGCATTTCTC; **(AS)** TACCCACCAGGACAACAAGG
***hOAT1***	**(S)** GCCTTCTTCATCTACTCCTGG; **(AS)** CCCGGAGTACCTCCATACT
***hOAT2***	**(S)** CCTCCAAGCTGCTGGTCTAC; **(AS)** CAGGTAGGCAGTGGTGAAGG
***hOAT3***	**(S)** GCTGAGCTGCCCTACTACAG; **(AS)** CGAGAAGGTCATGGCACTGG

Human embryonic kidney 293 wild type cell lines (HEK293) were seeded at the density of 5×10^5^ cell/well in six-well plates, and cultured for 24 h in a humidified incubator (37°C, 5% CO_2_). The spent culture medium was removed, and 2 ml of fresh medium containing DHI (1000, 500, 100, 50, and 20-fold dilution of DHI) or salvianolic acids (SaA, SaB, RA, DSS, PA, CA, and LA; 50 μM) was added to each well (n = 6). Finally, the cells were lysed with RNA lysis buffer after the incubation for 24 h, and the total RNA was collected for qPCR analysis. The primer sequences of *hOAT1*, *hOAT2*, and *hOAT3* were detailed in **Table 1**.

### Determination of the Effect of DHI on Transport Function of OATs

Transgenic HEK293 cell lines stably overexpressing OAT1 and OAT3 were cultured in high glucose DMEM containing 10% of fetal bovine serum, 1% of penicillin-streptomycin and 0.5 mg/ml of geneticin. The cells were seeded at the density of 1×10^6^ cell/well in 24-well plates, and transport experiments were performed when the cell density reached 80–90%. Spent culture medium was removed and cells were washed with pre-warmed HEPES buffer (50 mM of HEPES, 250 mM of NaCl, 9.6 mM of KCl, 11.2 mM of D-(+)-glucose, 2.4 mM of CaCl_2_, 2.4 mM of KH_2_PO4, and 2.4 mM of MgSO4; pH 7.4). Then the wash buffer was discarded and 0.25 ml of fresh HEPES buffer containing DHI and substrates was added to each well. There were eight concentrations of DHI prepared through diluting the original DHI by 2×10^4^, 1×10^4^, 2×10^3^, 1×10^3^, 500, 100, 20, and 10 times with physiological saline, respectively. The substrates of OAT1 and OAT3 were PAH (20 μM) and ES (20 μM), respectively. The transport system of positive control (PC) consisted of substrates and probenecid (20 μM), probenecid was a recognized inhibitor of both OAT1 and OAT3, while that blank control (CTL) containing only the substrates. Immediately after the incubation of 10 min, the cells were washed with pre-cooled HEPES buffer for three times, and then lysed with 0.15 ml of NaOH solution (1M) per well for 30 min at room temperature (25°C). When the cells were completely lysed, an equal amount of HCl solution (1M) was added to neutralize the lysate. An aliquot of the lysate was sampled to determine the protein concentration by Pierce^®^ BCA protein assay kit (ThermoFisher Scientific, Cat. No. 23225), and the remaining lysate was used to determine the concentration of PAH (Liu et al., 2012) and ES (Corona et al., 2010) by UHPLC-MS/MS. The uptake rates of OAT1 and OAT3 were calculated according to the concentrations of PAH and ES in lysate, respectively, which detailed as follows: Uptake rate (pmol/mg/min) = Substrate concentration/(Protein concentration × Incubation time).

### Experimental Design of Drug Interaction Between DHI and AAI

Twenty-four male SD rats weighting 350–400 g were randomly divided into two groups as follows (n = 12): rats in Group AAI were injected with physiological saline (4.16 ml/kg) through caudal vein 30 min before intraperitoneal injection of AAI (10 mg/kg); rats in Group AAI-DHI were injected with DHI (4.16 ml/kg) through caudal vein 30 min before intraperitoneal injection of AAI (10 mg/kg). The rate of caudal vein injection was controlled at about 1 ml/min. After the single administration of AAI and/or DHI, half of the rats in each group were sacrificed and kidneys were sampled for AAI quantification by UHPLC-MS/MS. The other half of the rats were used for blood collection at different time points (15, 30, 45, 60, 90, and 120 min after the injection of AAI), and the plasma samples were prepared for determination of AAI concentration by UHPLC-MS/MS.

Twenty-four male C57BL/6 mice weighting 25–30 g were randomly divided into three groups as follows (n = 8): mice in Group CTL were supplied with food and water regularly; mice in Group AAI were injected with AAI solution (10 mg/kg) intraperitoneally; mice in Group AAI-DHI were injected with DHI (6.01 ml/kg) through caudal vein 30 min before intraperitoneal injection of AAI (10 mg/kg). All mice were administrated daily at scheduled time (9:00–10:00 a.m.) for seven consecutive days. On the 8^th^ day, plasma was sampled from abdominal aorta of each mouse for the detection of creatinine level, and kidneys were collected and fixed in 10% of formalin buffer for hematoxylin-eosin staining (HE).

### Statistical Analysis

All data were expressed as means ± S.E.M. Differences among groups were analyzed by one-way ANOVA test. Statistical significant difference was set as *P*<0.05, and very significant difference was set as *P <*0.01.

## Results

### DHI Was Used in Combination With ASA With High Frequency in Clinic

A total of 5,183 clinical cases of DHI in Nanjing General Hospital of People’s Liberation Army from July 2012 to July 2017 were collected, and the analysis results revealed that 473 kinds of drugs might be used in combination with DHI for the treatment of cerebrovascular and cardiovascular diseases such as cerebral infarction and coronary heart disease. Among the 473 kinds of drugs, ASA was the drug that most commonly used in combination with DHI, and the frequency of drug combination was 70.73% (**Figure 1A**), which was in accordance with the conclusion reported in previous studies (Du et al., 2011). Top ten drugs that commonly used in combination with DHI were ASA, clopidogrel, oxiracetam injection, atorvastatin calcium, mecobalamin injection and tablet, rosuvastatin calcium, heparin sodium injection, lidocaine injection, fasudil injection, and phenobarbital injection.

### The Pharmacokinetics Profile of SA Was Influenced During Drug Combination of DHI and ASA

In the drug combination of DHI and ASA, the plasma concentration of SA was significantly increased at different time points (**Figure 1D**). The area under concentration-time curve (AUC_0-t_) and the maximum plasma concentration of SA (C_max_) was 2.57 and 2.37 times than that in single administration of ASA, respectively (**Figure 1D**; *P*<0.01).

In this herb-drug interaction experiment that performed on rats, DHI and ASA were administrated simultaneously, because as for 44.71% of the patients, the time interval between the use of DHI and ASA was counted to be zero according to the results of clinical case analysis (**Figure 1B**). The administration dose of DHI and ASA were designed based on the clinical conclusion reported previously (Chen et al., 2011), and the daily doses of DHI (4.16 ml/kg) and ASA (10.41 mg/kg) were equivalent to a 60 kg person taking 40 ml of DHI and 100 mg of ASA daily.

### DHI Increased the Plasma Concentration of SA Not Through Enhancing ASA Esterase Activity

**Figure 2:**
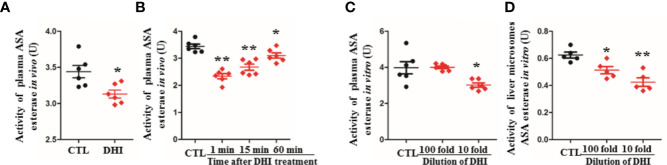
Inhibition potential of DHI on ASA esterase activity. **(A)** Activity of plasma ASA esterase *in vivo* when rats were treated with DHI for 14 consecutive days; **(B)** Activity of plasma ASA esterase *in vivo* at different time points (1, 5, and 60 min after DHI treatment); **(C)** Activity of plasma ASA esterase *in vitro*; **(D)** Activity of liver microsome ASA esterase *in vitro*. *P < 0.05, **P < 0.01.

The activity of plasma ASA esterase was significantly inhibited by 9.01% when rats were treated with DHI for 14 consecutive days (**Figure 2A**; *P* < 0.05), and the inhibition effect of DHI was fading when the plasma concentration of DHI was decreasing with time (**Figure 2B**; *P* < 0.05). Furthermore, the inhibitory effect of DHI on ASA esterase was confirmed *in vitro* study, as the activity of ASA esterase in plasma and liver microsome was significantly inhibited by DHI in a concentration dependent manner (**Figures 2C, D**, *P* < 0.05).

### Renal Excretion of SA Was Decreased During Drug Combination of DHI and ASA

**Figure 3:**
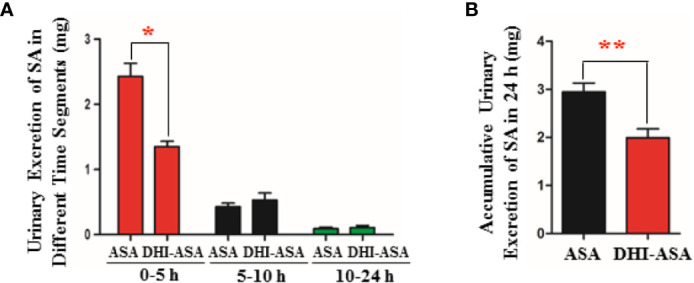
Decreased renal excretion of SA during drug combination of DHI and ASA. **(A)** Content of SA excreted in urine in different time segments; **(B)** Accumulative content of SA excreted in urine in 24 h. *P < 0.05, **P < 0.01.

When ASA was used in combination with DHI, the content of SA excreted in urine in the first five hours was significantly reduced by 44.44% from 2.43 ± 0.49 to 1.35 ± 0.20 mg (**Figure 3A**; *P* < 0.05). As to the accumulative urinary excretion of SA in 0–24 h, the content of SA was significantly decreased by 32.54% from 2.95 ± 0.45 to 1.99 ± 0.45 mg (**Figure 3B**; *P* < 0.01).

### The Tubular Reabsorption of SA Was Not Affected During Drug Combination of DHI and ASA

**Figure 4:**
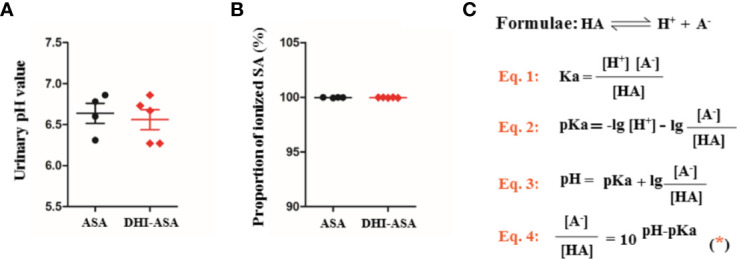
Tubular reabsorption of SA was not affected during drug combination of DHI and ASA; **(A)** pH value of urine; **(B)** Proportion of ionized SA; **(C)** Equations for calculating the proportion of ionized SA.

A slight decrease of urinary pH value by 0.08 was observed during drug combination of DHI and ASA as compared with single administration of ASA (**Figure 4A**). The proportion of ionized SA was further calculated based on the equation 4 (**Figure 4C**), which was obtained according to the acid-base proton theory (Equations 1 and 2) and the Henderson-Hasselbalch formula (Equation 3). The results showed that the SA was almost totally ionized in urine regardless of whether ASA was used alone or used in combination with DHI (**Figure 4B**), and thus the portion of SA reabsorbed by renal tubular is tiny.

### Gene Transcription and Protein Expression of OATs Were Down-Regulated by DHI

**Figure 5:**
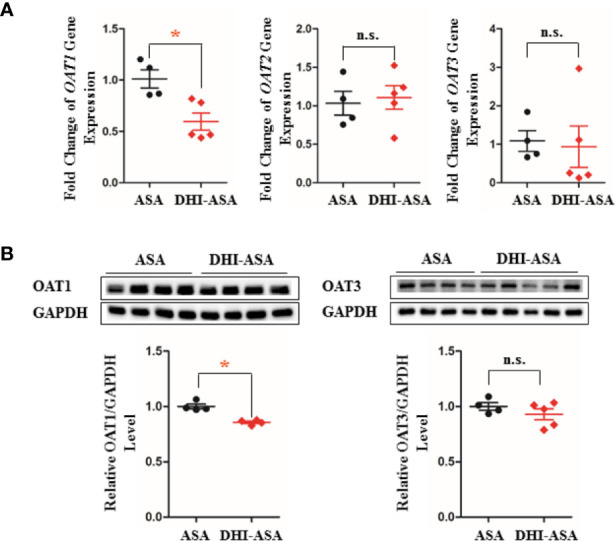
Inhibitory effect of DHI on OATs *in vivo*. **(A)** Gene transcription of OATs; **(B)** Protein expression of OATs during drug combination of DHI and ASA. *P < 0.05.

**Figure 6:**
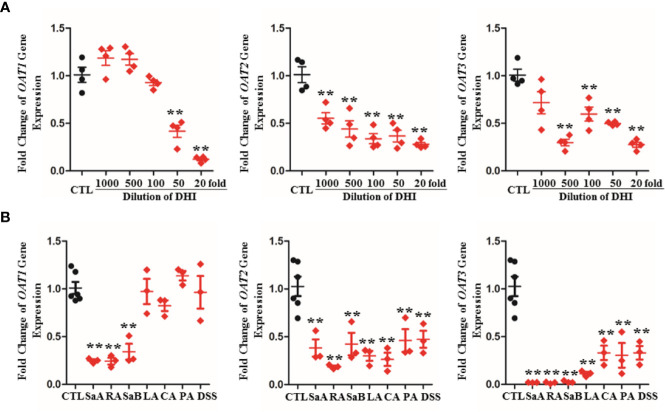
Inhibitory effect of DHI and salvianolic acids on OATs in HEK293 wild type cell lines. **(A)** Down-regulation of gene expression of *OAT1*, *OAT2*, and *OAT3* by DHI; **(B)** Down-regulation of gene expression of *OAT1*, *OAT2*, and *OAT3* by main salvianolic acids in DHI (salvianolic acid A, SaA; rosmarinic acid, RA; salvianolic acid B, SaB; lithospermic acid, LA; caffeic acid, CA; protocatechuic aldehyde, PA; Tanshinol, DSS). **P < 0.01.

The gene transcription and protein expression of OAT1 in kidney were significantly down-regulated during drug combination of DHI and ASA as compared to single administration of ASA (**Figures 5**, **S4**; *P* < 0.05). The inhibitory effect of DHI on OATs was further confirmed *in vitro* study, as the gene expression of *OAT1*, *OAT2*, and *OAT3* were significantly inhibited by DHI in HEK293 wild type cells (**Figure 6A**; *P* < 0.01).

To identify the components that contribute to the inhibitory potency of DHI, the effects of seven salvianolic acids on OATs gene expression were further analyzed. Salvianolic acids including SaA, SaB, RA, DSS, PA, CA, and LA are main bioactive components of DHI (Liu et al., 2013). The results showed that SaA, RA, and SaB significantly down-regulated the gene expression of *OAT1* by 75.21, 75.63, and 65.80%, respectively; SaA, RA, SaB, LA, CA, PA, and DSS significantly down-regulated the gene expression of *OAT2* by 61.79, 81.48, 57.66, 70.00, 73.66, 53.94, and 52.67%, and down-regulated the gene expression of *OAT3* by 98.02, 98.30, 97.27, 88.70, 66.95, 69.58, and 66.90%, respectively (**Figure 6B**; *P* < 0.01).

### Transport Function of OATs Was Inhibited by DHI

**Figure 7:**
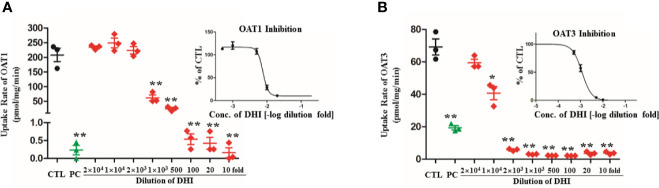
Inhibitory effect of DHI on transport function of OATs in transgenic HEK293 cells overexpressing *OAT1* and *OAT3*. **(A)** Uptake rate of PAH by OAT1; **(B)** Uptake rate of ES by OAT3. **P < 0.01.

DHI significantly inhibited the uptake of PAH by OAT1 and ES by OAT3 in transgenic HEK293 cells overexpressing *OAT1* and *OAT3*. The higher concentration of DHI, the stronger inhibitory effect on OAT transport function (**Figure 7**; *P* < 0.01). As to transport function of OAT1, even if the 1,000-fold dilution of DHI decreased the uptake of PAH by 70.35% from 208.00 to 61.67 pmol/mg/min (**Figure 7A**; *P* < 0.01). The inhibitory effect of DHI at high concentration (100, 20, and 10-fold dilution of DHI) was equivalent to that of positive control (probenecid, 20 μM). The inhibitory effect of DHI on transport function of OAT3 (IC50: 0.116% of DHI; 95% CI: 0.0991–0.137%; **Figure 7B**) was suggested to be stronger than that of OAT1 (IC50: 0.759% of DHI; 95% CI: 0.645–0.895%).

### DHI Inhibited OATs-Mediated AAI Transportation and Alleviated AAI Nephropathy

**Figure 8:**
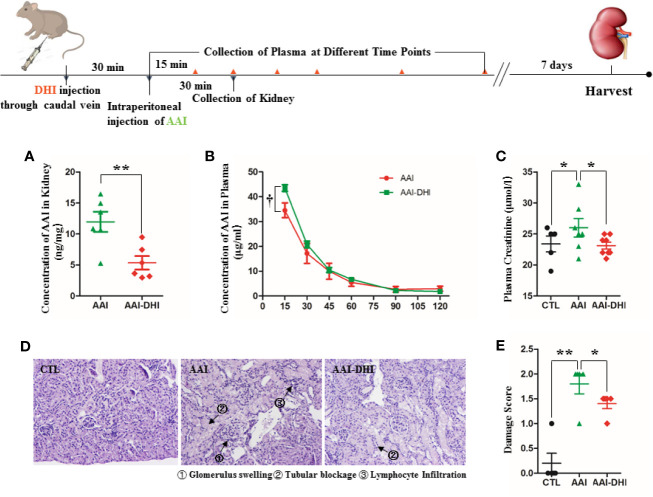
DHI decreased the renal accumulation of aristolochic acid I (AAI) and alleviated the renal damage induced by AAI. **(A)** Concentration of AAI in kidney; **(B)** Concentration of AAI in plasma; **(C)** Plasma concentration of creatinine; **(D)** Histological analysis of renal damage (HE staining, original magnification × 400); **(E)** Renal damage score. *P < 0.05, **P < 0.01.

When AAI was used in combination with DHI in rats, the AAI content in kidney was significantly decreased from 11.96 to 5.35 ng/mg (**Figure 8A**; *P* < 0.05). Accordingly, DHI significantly increased the plasma concentration of AAI at different time points, and the maximum concentration of AAI was increased from 34.54 to 43.51 μg/ml (**Figure 8B**; *P* < 0.05).

The renal damage induced by AAI in mice was mainly characterized as increased creatinine level (**Figure 8C**; *P* < 0.05), glomerulus swelling, tubular blockage, and lymphocyte infiltration (**Figure 8D**). When AAI was used in combination with DHI, the plasma level of creatinine was significantly decreased from 26.0 to 23.12 μmol/l (**Figure 8C**; *P* < 0.05). Furthermore, the glomerulus swelling and lymphocyte filtration were significantly alleviated, and the total damage score was reduced from 1.8 to 1.4 (**Figures 8D, E**; *P* < 0.05).

## Discussion

**Figure 9:**
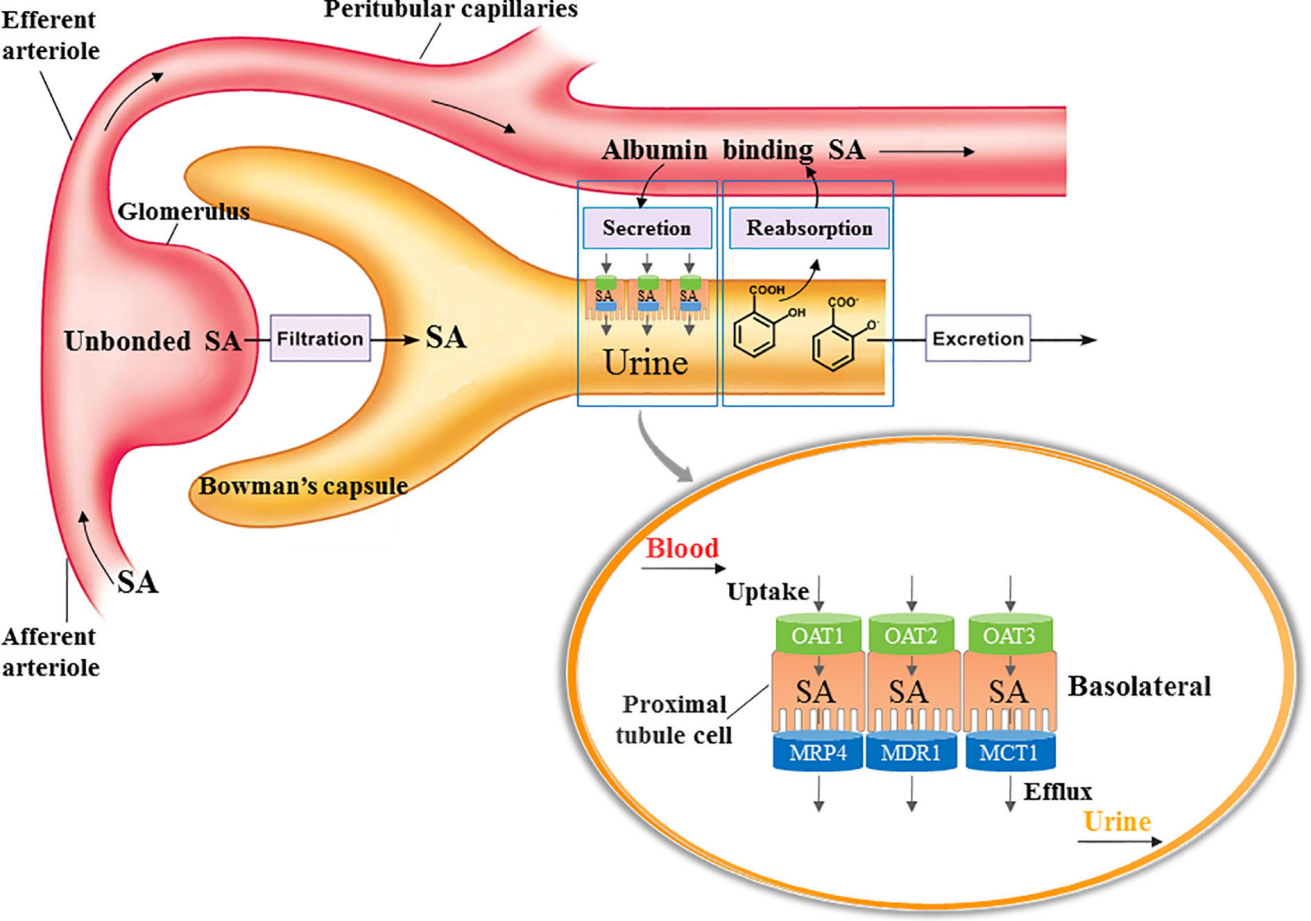
Process of SA excretion in kidney. Glomerular filtration, tubular secretion and reabsorption are primary processes of renal excretion of SA. The SA in blood circulation enters the glomerulus through afferent arteriole, and the unbound SA is directly filtered into the Bowman’s capsule. The SA bound with albumin would be flowed into peritubular capillaries and transported from plasma into proximal tubular cells by OATs which are located in the basolateral membrane of proximal tubular cells. Then SA in the proximal tubular cells would be excreted into the lumen of tubule by efflux transporters such as multidrug resistance protein 4 (MRP4), multi drug resistance transporter 1 (MDR1), and mono carboxylate transporter 1 (MCT1), which are located in the apical membrane of proximal tubule cells. The molecular form of SA in the urine would be reabsorbed back into peritubular capillaries, while the SA in ionic form would be excreted with urine.

Glomerular filtration, tubular secretion and reabsorption are primary processes of renal excretion of SA (**Figure 9**). The SA in blood circulation enters the glomerulus through afferent arteriole, and the unbound SA is directly filtered into the Bowman’s capsule. The SA bound with albumin would be flowed into peritubular capillaries and transported from plasma into proximal tubular cells by OATs which are located in the basolateral membrane of proximal tubular cells. Then SA in the proximal tubular cells would be excreted into the lumen of tubule by efflux transporters such as multidrug resistance protein 4 (MRP4; Mattiello et al., 2011), multi drug resistance transporter 1 (MDR1; Polachek et al., 2010), and mono carboxylate transporter 1 (MCT1; Tamai et al., 1999), which are located in the apical membrane of proximal tubule cells. The molecular form of SA in the urine would be reabsorbed back into peritubular capillaries, while the SA in ionic form would be excreted with urine (Weiner et al., 1959; Miners, 1989).

In drug combination of DHI and ASA, the renal excretion of SA was significantly decreased, which might be caused by the alteration of SA excretion through glomerular filtration, tubular secretion or tubular reabsorption. Our results suggested that the tubular reabsorption of SA was not affected during drug combination of DHI and ASA. According to the consensus of ion trapping, the tubular reabsorption of SA is primarily regulated by the acidity-alkalinity of urine (Proudfoot et al., 2003). Because SA is weakly ionized in physiological fluids, and only the unionized SA with high fat solubility can be absorbed through the tubule epithelium and the capillary endothelium. Our data showed a slight decrease of urinary pH value when DHI was used in combination with ASA. However, SA was almost totally ionized in urine regardless of whether ASA was used alone or used in combination with DHI. Therefore, the portion of SA reabsorbed by renal tubular is tiny.

Salvianolic acids in DHI are considered as strong ligands of albumin, and the binding ability to human serum albumin of salvianolic acids are stronger than sodium salicylate (Zhu et al., 2017). It is possible that the SA bound with albumin in blood circulation might be squeezed down by salvianolic acids, and the proportion of SA directly excreted by glomerular filtration might be consequently increased. Our data showed that the renal excretion of SA was significantly decreased during drug combination of DHI and ASA, which suggested that the alteration of SA excretion by glomerular filtration might not be the main contributor.

OATs are suggested to be central to the renal tubular secretion of SA (**Figure 9**; Bow et al., 2006; Emami Riedmaeir et al., 2012; Nigam et al., 2015). OATs belong to the solute carrier 22 subfamily (SLC22) of major facilitator superfamily (MFS), and are generally considered as influx transporters that facilitate the movement of substrates from blood circulation into the renal tubular epithelial cells (Nigam et al., 2015). OATs are currently recognized as the most important membrane transporters and have been demonstrated to be the important mediator of many DDIs (Emami Riedmaeir et al., 2012). Our results showed that the gene transcription, protein expression, and transport function of OATs were significantly down-regulated by DHI, which might consequently induce the decrease of SA renal excretion during drug combination of DHI and ASA. However, the limitation was that the molecular biotechnology applied in this section such as western blot was not sensitive enough to reveal the small change of OATs, and the methods with better sensitivity and reliability such as targeted quantitative proteomics, as well as application of probe substrates would be applied to confirm the effect of DHI on OATs in our future study. Efflux transporters located in the apical membrane of proximal tubule cells are also critical in renal tubular secretion of SA, because MRP4, MDR, and MCT1 facilitate the transportation of SA from proximal tubule cells to tubule lumen. However, our results showed that no significant changes of gene transcription of *MRP4*, *MDR1*, or *MCT1* were observed in drug combination of DHI and ASA (**Figure S3**; **Table S1**).

OATs play a key role in the pathogenesis of AAI nephropathy, previous studies have confirmed that the transportation of AAI from blood circulation to proximal tubular cells by OAT1 and OAT3 is the initial cause of renal damage induced by AAI (Bakhiya et al., 2009; Xue et al., 2011), and the acute tubular injuries induced by AAI can be alleviated by probenecid, an inhibitor of OATs (Baudoux et al., 2012). In order to confirm the inhibitory effect of DHI on OATs *in vivo*, we further studied whether DHI could inhibit OATs-mediated AAI transportation into kidney and alleviate AAI induced nephropathy. Our results showed that DHI significantly reduced the renal accumulation of AAI, and alleviated renal damage such as glomerulus swelling, tubular blockage and lymphocyte filtration.

ASA esterase (Enzyme Commission number: 3.1.1.55) is the general term of a kind of hydrolase that acts on the ester bond to hydrolyze ASA, which is also known as acetylsalicylic deacetylase (Braunschweig Enzyme Database, BRENDA). The hydrolysis process of ASA to SA is principally determined by ASA esterase activity in addition to the weak role of simple hydrolysis. Therefore, the production of SA released from ASA is primarily determined by ASA esterase activity (White et al., 2005). In drug combination of DHI and ASA, the plasma concentration of SA was significantly increased. It is possible that the increase of SA plasma concentration was caused by the enhanced activity of ASA esterase. However, our results suggested that the activity of ASA esterase was significantly inhibited by DHI, and the alteration of ASA esterase activity might not be the reason that contribute to the increase of SA concentration.

In this study, hydrolysis of ASA regulated by ASA esterase, tubular secretion of SA mediated by influx and efflux transporters, and tubular reabsorption of SA regulated by urinary acidity-alkalinity were included in the mechanism study of herb-drug interaction between DHI and ASA. However, there are still other possible mechanisms that have not been reported. For example, in blood circulation, a portion of SA remains the prototype, and the other portion of SA is metabolized to salicyluric acid, gentisic acid, salicyl acyl glucuronide, salicyl phenolic glucuronide, salicyluric acid phenolic glucuronide and gentisuric acid by cytochrome P450, acyl-CoA N-acyltransferase or uridine 5’-diphosphoglucuronosyltransferases (Li et al., 2017). Each metabolite should undergo a complete and independent process of renal excretion. For example, salicyluric acid is also excreted mainly by glomerular filtration, tubular secretion and absorption (Cox et al., 1989). The results regarding the influence of drug combination on renal excretion of other metabolites such as salicyluric acid, gentisic acid, salicyl phenolic glucuronide and salicyluric acid phenolic glucuronide would be presented in our another manuscript. Furthermore, SA can be metabolized to 2,3-dihydroxybenzoic acid by direct hydroxyl radical attack, and the plasma level of 2,3-dihydroxybenzoate is suggested to be powerful biomarker of oxidative stress in chronic complications (Ghiselli et al., 1992). This is a new insight of herb-drug interaction between DHI and ASA, which would be included in our future study.

## Conclusion

ASA and DHI were widely used in combination in clinic for the treatment of cardiovascular and cerebrovascular diseases. The drug-herb interaction between ASA and DHI was characterized by increased plasma concentration of SA, and decreased renal excretion of SA. The inhibitory effect of DHI on the gene transcription, protein expression and transport function of OATs might be the reason that contribute to the decrease of SA renal excretion.

## Data Availability Statement

The original contributions presented in the study are included in the article/supplementary material; further inquiries can be directed to the corresponding authors.

## Ethics Statement

The experiments on rats were approved by the Animal Ethics Committee of Nanjing University of Chinese medicine, and performed in compliance with the Guide for the Care and Use of Laboratory Animals.

## Author Contributions

JPL, JG, and JD designed research. JPL, JBL, YP, XX, and CC performed research. MG and LL analyzed clinical cases. JPL wrote the paper. All authors contributed to the article and approved the submitted version.

## Funding

This work was supported by the National Natural Science Foundation of China (Grant numbers 81803795); Natural Science Foundation of Jiangsu Province of China (Grant number BK20180823); and Natural Science Foundation of the Jiangsu Higher Education Institutions of China (Grant number 18KJB360003).

## Conflict of Interest

The authors declare that the research was conducted in the absence of any commercial or financial relationships that could be construed as a potential conflict of interest.

## References

[B1] Bakhiya N. Arlt V. M. Bahn A. Burckhardt G. Phillips D. H. Glatt H. (2009). Molecular evidence for an involvement of organic anion transporters (OATs) in aristolochic acid nephropathy. Toxicol 264, 74–79. 10.1016/j.tox.2009.07.014 19643159

[B2] Baudoux T. E. Pozdzik A. A. Arlt V. M. De Prez E. G. Antoine M. H. Quellard N. (2012). Probenecid prevents acute tubular necrosis in a mouse model of aristolochic acid nephropathy. Kidney Int. 82, 1105–1113. 10.1038/ki.2012.264 22854641

[B3] Bow D. A. Perry J. L. Simon J. D. Pritchard J. B. (2006). The impact of plasma protein binding on the renal transport of organic anions. J. Pharmacol. Exp. Ther. 316, 349–355. 10.1124/jpet.105.093070 16195420

[B4] Chen Q. Yi D. H. Xie Y. M. Yang W. Yang W. Yang W. (2011). Analysis of clinical use of Danhong injection based on hospital information system. Zhongguo Zhong Yao Za Zhi 36, 2817–2820. 10.4268/cjcmm20112016 22292374

[B5] Corona G. Elia C. Casetta B. Da Ponte A. Del Pup L. Ottavian E. (2010). Liquid chromatography tandem mass spectrometry assay for fast and sensitive quantification of estrone-sulfate. Clin. Chim. Acta 411, 574–580. 10.1016/j.cca.2010.01.019 20096278

[B6] Cox P. G. Moons W. M. Russel F. G. Ginneken C. A. (1989). Renal handling of salicyluric acid in the isolated perfused rat kidney: evidence for accumulation in tubular cells. J. Pharmacol. Exp. Ther. 251, 750–755. 2810125

[B7] Diehl K. H. Hull R. Morton D. Pfister R. Rabemampianina Y. Smith D. (2001). A good practice guide to the administration of substances and removal of blood, including routes and volumes. J. Appl. Toxicol. 21, 15–23. 10.1002/jat.727 11180276

[B8] Du J. Yang W. Yi D. H. Xie Y. M. Yang W. Yang W. (2011). Analysis of using Danhong injection to treatment coronary heart disease patient’s medicines based on real world HIS database. Zhongguo Zhong Yao Za Zhi 36, 2821–2824. 10.4268/cjcmm20112017 22292375

[B9] Emami Riedmaier A. Nies A. T. Schaeffeler E. Schwab M. (2012). Organic anion transporters and their implications in pharmacotherapy. Pharmacol. Rev. 64, 421–449. 10.1124/pr.111.004614 22457399

[B10] Ghiselli A. Laurenti O. Mattia G. D. Maiani G. Ferro-Luzzi A. (1992). Salicylate hydroxylation as an early marker of in vivo oxidative stress in diabetic patients. Free Radic. Biol. Med. 13, 621–626. 10.1016/0891-5849(92)90036-g 1459481

[B11] Li J. P. Guo J. M. Hua Y. Q. Zhu K. Y. Tang Y. P. Zhao B. C. (2016a). The mixture of Salvia miltiorrhiza-Carthamus tinctorius (Danhong injection) alleviates low-dose aspirin induced gastric mucosal damage in rats. Phytomedicine 23, 662–671. 10.1016/j.phymed.2016.03.006 27161407

[B12] Li J. P. Guo J. M. Shang E. X. Zhu Z. H. Zhu K. Y. Li S. J. (2016b). A metabolomics strategy to explore urinary biomarkers and metabolicpathways for assessment of interaction between Danhong injection and low-dose aspirin during their synergistic treatment. J. Chromatogr. B. 1026, 168–175. 10.1016/j.jchromb.2015.07.045 26265434

[B13] Li J. P. Guo J. M. Shang E. X. Zhu Z. H. Liu Y. Zhao B. C. (2017). Quantitative determination of five metabolites of aspirin by UHPLC-MS/MS coupled with enzymatic reaction and its application to evaluate the effects of aspirin dosage on the metabolic profile. J. Pharm. Biomed. Anal. 138, 109–117. 10.1016/j.jpba.2016.12.038 28192718

[B14] Liu T. Meng Q. Wang C. Liu Q. Guo X. Sun H. (2012). Changes in expression of renal Oat1, Oat3 and Mrp2 in cisplatin-induced acute renal failure after treatment of JBP485 in rats. Toxicol. Appl. Pharmacol. 264, 423–430. 10.1016/j.taap.2012.08.019 22992436

[B15] Liu X. Wu Z. Yang K. Ding H. Wu Y. (2013). Quantitative analysis combined with chromatographic fingerprint for comprehensive evaluation of Danhong injection using HPLC-DAD. J. Pharm. Biomed. Anal. 76, 70–74. 10.1016/j.jpba.2012.12.013 23298908

[B16] Mattiello T. Guerriero R. Lotti L. V. Trifiro E. Felli M. P. Barbarulo A. (2011). Aspirin extrusion from human platelets through multidrug resistance protein-4-mediated transport: Evidence of a reduced drug action in patients after coronary artery bypass grafting. J. Am. Coll. Cardiol. 58, 752–761. 10.1016/j.jacc.2011.03.049 21816313

[B17] Miners J. O. (1989). Drug interactions involving aspirin (acetylsalicylic acid) and salicylic acid. Clin. Pharmacokinet. 17, 327–344. 10.2165/00003088-198917050-00003 2573442

[B18] Nigam S. K. Bush K. T. Martovetsky G. Ahn S. Y. Liu H. C. Richard E. (2015). The organic anion transporter (OAT) family: a systems biology perspective. Physiol. Rev. 95, 83–123. 10.1152/physrev.00025.2013 25540139 PMC4281586

[B19] Polachek H. Holcberg G. Polachek J. Rubin M. Feinshtein V. Sheiner E. (2010). Carrier-mediated uptake of levofloxacin by bewo cells, a human trophoblast cell line. Arch. Gynecol. Obstet. 281, 833–838. 10.1007/s00404-009-1177-y 19629508

[B20] Proudfoot A. T. Krenzelok E. P. Brent J. Vale J. A. (2003). Does urine alkalinization increase salicylate elimination? If so, why? Toxicol. Rev. 22, 129–136. 10.2165/00139709-200322030-00001 15181662

[B21] Robbins N. Koch S. E. Tranter M. Rubinstein J. (2012). The history and future of probenecid. Cardiovasc. Toxicol. 12, 1–9. 10.1007/s12012-011-9145-8 21938493

[B22] Schmassmann-Suhijar D. Bullingham R. Gasser R. Schmutz J. Haefeli W. E. (1998). Rhabdomyolysis due to interaction of simvastatin with mibefradil. Lancet 351, 1929–1930. 10.1016/S0140-6736(05)78613-X 9654265

[B23] Tamai I. Sai Y. Ono A. Kido Y. Yabuuchi H. Takanaga H. (1999). Immunohistochemical and functional characterization of ph-dependent intestinal absorption of weak organic acids by the monocarboxylic acid transporter mct1. J. Pharm. Pharmacol. 51, 1113–1121. 10.1211/0022357991776804 10579682

[B24] Weiner I. M. Washington J. A. Mudge G. H. (1959). Studies on the renal excretion of salicylate in the dog. Bull. Johns Hopkins Hosp. 105, 284–297. 13843574

[B25] White S. Calver B. L. Newsway V. Wade R. Patel S. Bayer A. (2005). Enzymes of drug metabolism during delirium. Age Ageing 34, 603–608. 10.1093/ageing/afi189 16267186

[B26] Xue X. Gong L. K. Maeda K. Luan Y. Qi X. M. Sugiyama Y. (2011). Critical role of organic anion transporters 1 and 3 in kidney accumulation and toxicity of aristolochic acid I. Mol. Pharm. 8, 2183–2192. 10.1021/mp100418u 21980933

[B27] Zhu J. F. Yi X. J. Huang P. Chen S. Q. Wu Y. J. (2017). Drug-protein binding of Danhong injection and the potentialinfluence of drug combination with aspirin: Insight by ultrafiltration LC-MS and molecular modeling. J. Pharm. Biomed. Anal. 134, 100–107. 10.1016/j.jpba.2016.11.028 27889668

