# Supplementary material for: Characteristic and Mechanism of Drug-Herb Interaction Between Acetylsalicylic Acid and Danhong Injection Mediated by Organic Anion Transporters
Source: Front Pharmacol. 2020 Oct 2;11:577012. doi: 10.3389/fphar.2020.577012 (PMC7562828; doi:10.3389/fphar.2020.577012)
Supplement: Supplementary file 2 [file DataSheet_2.pdf]

Methods of characterizing DHI

The chromatographic fingerprint of DHI and the quantification of salvianolic acid A (SaA), salvianolic acid B (SaB), rosmarinic acid (RA), tanshinol (danshensu, DSS), protocatechuic aldehyde (PA), caffeic acid (CA) and lithospermic acid (LA) was performed on ultra-high-performance liquid chromatography coupled with photo-diode array and quadrupole time of flight mass spectrometry (UHPLC-PDA-QTOF/MS; Waters Corp., Milford, USA). The separation was conducted on an Acquity UPLC BEH C 18 column (100 mm ×2.1 mm, 1.7 μm; Waters), and the mobile phase consisted of water-formic acid (A; 100:0.1, v/v) and acetonitrile (B). The conditions of gradient eluting were optimized as follows: 5-40% B (0-9.0 min), 40-80% B (9.0-10.0 min), 80-80% B (10.0-12.0 min), 80-5% B (12.0-12.5 min). The flow rate was 0.4 ml/min and the injection volume was 1 μl. The analytical method of quantification was validated by linearity, recovery, inter-day and intra-day precision, and short-term stability.

Figure legends

Supplementary Figure 1 Typical UPLC-PDA fingerprint BPI chromatogram of DHI (Lot Number: 13042014) and chromatographic peaks for salvianolic acid A (SaA), salvianolic acid B (SaB), rosmarinic acid (RA), tanshinol (danshensu, DSS), protocatechuic aldehyde (PA), caffeic acid (CA) and lithospermic acid (LA).

Supplementary Figure 2 Chemical structures of salvianolic acid A (SaA), salvianolic acid B (SaB), rosmarinic acid (RA), tanshinol (danshensu, DSS), protocatechuic aldehyde (PA), caffeic acid (CA) and lithospermic acid (LA).

Supplementary Figure 3 Gene transcription levels of *MRP4*, *MDR1* and *MCT1*.

Supplementary Figure 4 CT value of *β-Actin* gene amplification in qPCR analysis when ASA was used in combination with DHI in rats.

Supplementary Table 1 Primers of MCT1, MDR1 and MRP4

Primer	Sequence (5'-3')
MCT1-S	GTATGCCGGAGGTCCTATC
MCT1-AS	AAGCTGCAATCAAGCCACAG
MRP4-S	CCGACACTCAGGAACCGAAC
MRP4-AS	TTCTCTGCATCTTGGGCATCTG
MDR1-S	CGTCATCGTGGAGCAAGGAA
MDR1-AS	ATTGGTTTCCACATCCAGCCT
